# Predictive modeling of signal-responsive *cis*-elements in human red blood cell precursors

**DOI:** 10.1093/nar/gkag505

**Published:** 2026-05-21

**Authors:** Venkatasai Rahul Dogiparthi, Linda Chee, Pooja Roy, Yichao Zhou, M Jordan Rowley, Kyle J Hewitt

**Affiliations:** Department of Genetics, Cell Biology and Anatomy, University of Nebraska Medical Center, 985805 Nebraska Medical Center, Omaha, NE 68124, United States; Department of Genetics, Cell Biology and Anatomy, University of Nebraska Medical Center, 985805 Nebraska Medical Center, Omaha, NE 68124, United States; Department of Genetics, Cell Biology and Anatomy, University of Nebraska Medical Center, 985805 Nebraska Medical Center, Omaha, NE 68124, United States; Department of Genetics, Cell Biology and Anatomy, University of Nebraska Medical Center, 985805 Nebraska Medical Center, Omaha, NE 68124, United States; Department of Genetics, Cell Biology and Anatomy, University of Nebraska Medical Center, 985805 Nebraska Medical Center, Omaha, NE 68124, United States; Department of Genetics, Cell Biology and Anatomy, University of Nebraska Medical Center, 985805 Nebraska Medical Center, Omaha, NE 68124, United States

## Abstract

Signal-responsive transcriptional programs are turned on/off by *cis*-regulatory elements (*cis*-elements) acting in coordination with gene promoters. While large genomics datasets have annotated common *cis*-element features, interpreting and predicting specific environmental responses at the chromatin level are critical ongoing challenges. We combined signal-induced chromatin accessibility changes with transcriptomics and a comprehensive catalogue of chromatin occupancy features for predicting functional signaling responses. Using the Kit receptor tyrosine kinase pathway—a central pro-survival, pro-proliferation, and pro-differentiation pathway in hematopoiesis, erythropoiesis, and other tissues—we mapped the sequence and chromatin features at thousands of Kit signal-responsive *cis*-elements in the human genome. A subset of Kit-induced changes to chromatin occupancy required upregulation of early growth response-1 (EGR1), uncovering distinct EGR1-sensitive and EGR1-insensitive branches of Kit signaling. Predictions of chromatin features associated with signal responses were tested using CRISPR-mediated *cis*-element disruption, which impaired target gene activation even at very long ranges. These findings define subsets of Kit signaling-dependent *cis*-regulatory logic which can be applied to interpret how growth factor pathways and cell-type specific transcription factors communicate on chromatin to direct cell behaviors in normal and disease contexts.

## Graphical Abstract

**Graphical Abstract:**
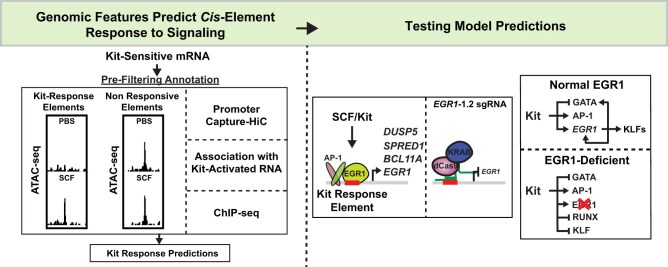

## Introduction

*Cis*-regulatory DNA elements are interfaces between signaling cascades and the transcriptional machinery. By integrating diverse, dynamic, and often opposing signaling inputs, *cis*-elements modulate transcriptional output to preserve cell identity and maintain homeostasis within cellular niches [1, 2]. Signal-dependent transcriptional regulation via *cis*-elements is a fundamental regulatory program governing normal development and disease states. For example, Activator Protein-1 (AP-1) transcription factors are rapidly induced by growth factor signaling and cooperate with nuclear receptors/cofactors to drive changes in chromatin accessibility [3, 4]. Similarly, Signal Transducer and Activator of Transcription (STAT) proteins are key mediators of inflammatory and stress-induced gene expression programs [5]. Developmental pathways including bone morphogenetic protein and Wingless (Wnt) signaling, coordinate with lineage-specific transcription factors to establish cell type-specific programs and influence cell behaviors [6, 7]. Disruption of signal-responsive *cis*-element motifs, e.g. at the intersectin-1 (*ITSN1*) gene locus, impairs gene expression and correlates with selective change to transcription factor occupancies [6]. Signal-responsive *cis*-elements are enriched for single nucleotide polymorphisms (SNPs) associated with phenotypic variation in human populations [6, 8], suggesting that sequence-level information at signal-responsive *cis*-elements may help to forecast disease and treatment outcomes.

The chromatin occupancy of cell type-specific proteins and histone modifications at individual *cis*-elements provide critical insights into mechanisms of transcriptional activity [9]. Given the vast quantity of data that map chromatin features and structural organization of the genome [10], it is in principle possible to predict transcriptional outputs in response to signaling pathway activation. Indeed, chromatin features at *cis*-elements can predict the function of individual DNA elements in normal physiological processes, including cell fate decisions and differentiation [11–14]. Prediction models classify *cis*-elements as promoters, enhancers, or repressed regions across the genome, allowing comparative analyses across cell types under steady-state conditions, e.g. [15, 16]. However, chromatin accessibility and protein occupancy at specific *cis*-elements are dynamically responsive to signaling pathways, which complicates interpretation. While prediction models can identify basal *cis*-element activities at some sites, it is important to distinguish signaling-dependent transcriptional responses of *cis*-elements, especially when signaling inputs that alter cell behavior are transient, overlapping, or combinatorial.

Kit receptor tyrosine kinase (RTK) signaling is a well-studied pathway that converges on STAT5-, ERK-, and PI3K-dependent factors to control transcriptional programs involved in stem/progenitor cell functions [17, 18]. In the blood system, Kit signaling governs hematopoietic stem/progenitor cell survival, proliferation, and differentiation through lineage-restricted transcriptional regulators, including GATA2 [19, 20]. Dysregulation of Kit signaling has been implicated in various hematologic disorders, including anemia and certain leukemias [21]. In anemia contexts, Kit pathway activation stimulates erythroid progenitor activities and regeneration [22–24]. The balanced production of hematopoietic cells requires signaling crosstalk between Kit and other signaling pathways [e.g. erythropoietin (Epo) in erythroid precursors] at distinct stages of differentiation [25]. Kit pathway transcriptional targets in hematopoiesis include the *B-cell lymphoma-xL* (*BCL-xL*) gene involved in survival and the early growth response-1 (EGR1) transcription factor [18, 26]. Since many of the stress-dependent and/or clinically-relevant *cis*-elements in erythroid precursor cells are evolutionarily conserved and associated with important cell functions [8, 27, 28], unravelling *cis*-element signaling responsiveness genome-wide may uncover unique mechanisms and therapeutic targets.

Distinguishing whether accessibility or activity changes will be driven by a signaling input or by intrinsic chromatin context is a complex problem. Our study used aggregate publicly available datasets in static Kit-expressing hematopoietic cells and dynamic data we generated to profile all accessible *cis*-elements genome wide and predict their signal-dependent activity. Despite finding that the Kit pathway utilizes diverse mechanisms to activate transcription, activity can be predicted with high accuracy and provides unique profiles of hundreds of new genetic regulators in red blood cell precursors.

## Materials and methods

### Cell culture

Human umbilical cord blood-derived erythroid progenitor 2 (HUDEP-2) cells were obtained from Yukio Nakamura’s lab [29]. HUDEP-2 were grown at densities between 0.2 and 1.0 × 10^6^/ml in StemSpan Serum Free Expansion Media (SFEM) (StemCell Technologies) and supplemented with 2% penicillin/streptomycin (P/S), 0.4 μg/ml dexamethasone, 50 ng/ml recombinant human (rh) stem cell factor (SCF), 4 U/ml EPO, and 1 μg/ml doxycycline. For acute signaling assays, cells were grown in StemSpan SFEM (StemCell Technologies) supplemented with 2% P/S and 1% bovine serum albumin (BSA) for 5 h and then stimulated with 50 ng/ml recombinant human SCF (R&D Biosystems) for 1 h.

CD34^+^ cells were harvested from G-CSF-mobilized human peripheral blood purchased from Fred Hutch Comprehensive Center for Excellence in Hematology. The cells were thawed and maintained at 2 × 10^5^ per ml for 4 days in StemSpan SFEM supplemented with 1X CC100 cytokine mix (Flt-3 ligand, SCF, IL-3, IL-6). For erythroid lineage differentiation, cells were replated in Iscove’s Modified Dulbecco’s Medium supplemented with 15% fetal bovine serum (FBS), 2 mM glutamine, 1% BSA, 500 µg/ml holo human transferrin, 10 µg /ml rh insulin, 1 µM dexamethasone, 1 µM β-estradiol, 5 ng/ml IL-3, 100 ng/ml SCF, and 6U EPO) for 8 days. Cells were Kit^+^ selected using biotin-conjugated anti-human CD117 antibody (Biolegend) followed by streptavidin-coated MojoSort magnetic beads (BioLegend) and then harvested for ATAC-seq and RNA-seq.

### Lentiviral transfections, infections, and nucleofections

Single guide RNAs (sgRNAs) were cloned into pL-CRISPR.EFS.GFP (Addgene #57818) for enhancer knockouts and Lenti-(BB)-EF1a-KRAB-dCas9-P2A-EGFP (Addgene #71237) (Supplementary Table S1) prior to making lentiviral particles in human embryonic kidney 293T (HEK-293T) cells. One million HUDEP-2 cells were infected with 400 µl of virus containing polybrene (8 μg/ml) and 100× HEPES (5 μl per infection), spinoculated at 2600 rpm for 90 min, and GFP-sorted 48 h later. Alternatively, enhancer knockout cells were generated by nucleofection of ribonucleoprotein complexes. Two sgRNAs (Synthego) mixed with 20 μM Cas9 and 250 000 HUDEP-2 were nucleofected using the 4D-nucleofector™ X (cat. AAF-1003X, Lonza, Basel, Switzerland). Cells were maintained for 48–72 h for recovery in expansion media. To evaluate editing efficiencies, HUDEP-2 cells were screened for deletions/mutations by polymerase chain reaction (PCR) amplification of the surrounding region followed by either T7 endonuclease I digestion (1 unit for 15 min) followed by agarose gel electrophoresis, Sanger sequencing, and/or quantitative PCR (qPCR) using site-specific primers. Motifs at/nearby edited KREs were identified by MoLoTool web interface [30].

### Apoptosis

HUDEP-2 cells infected with sgControl and sgEGR1 constructs were observed for expansion defects by daily cell counts. Cells were stained using a Pacific Blue-conjugated Annexin V antibody for 15 min at 4°C. Cells were resuspended with Draq7 viability dye as required and analyzed using an Attune flow cytometer (Thermo Fisher). Data were analyzed with FlowJo v10.6.2.

### ATAC-seq and analysis

ATAC-seq was conducted using 50 000 cells per condition as biological duplicates according to [31]. Nuclei of lysed cells were treated with Tn5 enzyme containing transposition reaction mix using Nextera DNA Library Prep Kit (NEB). DNA purification was done using the Qiagen MinElute Reaction Cleanup Kit, and adapters were added to Tn5-treated samples for library preparation by using the kit NEBNext Ultra II Q5 2× Master Mix (NEB). Libraries were amplified by PCR and quantified using the QuantiFluor ONE double-stranded DNA System and High Sensitivity DNA ScreenTape Analysis, and all libraries were sequenced paired end (2 × 75 bp) on an Illumina NovaSeq X instrument. Raw data were analyzed in the ENCODE ATAC-seq pipeline mapped to hg38 human genome build. MAnorm was used to identify differentially accessible sites as described [32]. Motif enrichment was evaluated using HOMER [33]. ATAC-seq footprints were identified by TOBIAS as described in [34] Individual comparisons were performed against SCF- and PBS-treated samples in both sgControl and sgEGR1 to identify both Kit-sensitive and EGR1-sensitive footprints. SNPs with hematopoietic traits were identified at Kit-activated and Kit-repressed regions by genetic-chromVAR (gChromVAR) tool [35].

### RNA-seq and differential gene expression analysis

RNA-seq was conducted on 1 × 10^6^ HUDEP-2 and primary human erythroid cells derived from CD34+ progenitors individually-isolated in TriZol lysis buffer (Ambion) as biological replicates (*N* = 3 per condition). The extracted RNA was purified using Zymo RNA Clean & Concentrator-5 columns. Strand-specific RNA-seq libraries were prepared using the TruSeq library preparation kits and paired-end sequenced (2 × 75 bp) on an Illumina NextSeq 550 platform. Raw FASTQ files were obtained from UNMC Sequencing Core Facility. HISAT 2.1.0 aligner was used to align the sequences to the human genome hg38 assembly [36]. StringTie 2.1.1 was used to generate gene and transcript counts matrices [37, 38]. DESeq2 library from R Bioconductor identified the differentially regulated genes from normalized read counts [39]. Volcano plots were generated in GraphPad Prism.

### Prefiltering criteria and annotation

Promoters of differentially expressed genes (fold change > 0, p-adj < 0.05) from RNA-seq were identified and marked. Promoter-capture HiC (PC-HiC) data were collected from primary erythroblasts cells [40], and the bait regions were mapped to Kit target gene promoters to generate a list of putative Kit-interacting *cis*-elements. Accessible regions nearby Kit target gene promoters (<15 kb) were also included. Genome-wide activity by contact (ABC) scores in K562 cells [41] were annotated to putative *cis*-elements. Protein occupancy data were collected from 376 ChIP-seq data sets from K562, HUDEP-2, and primary erythroblast cells in ENCODE Consortium and ChIP-Atlas [42, 43]. After eliminating poor-quality datasets and datasets with no occupancy (Supplementary Table S2), 286 ChIP-seq datasets were annotated to putative *cis*-elements. Replicate ChIP-seq datasets for the same protein were merged.

### Model training

Transcription factor occupancy at KREs was determined by intersecting the peak calls from individual ChIP-seq datasets, and overlapped regions were merged. The filtered dataset was trained on XGBoost classifier model [44]. Class imbalances were addressed by performing the Synthetic Minority Oversampling Technique. L1 and L2 regularization has been incorporated to enhance the prediction capability and prevent overfitting. We trained the model using transcription factor occupancy at KREs. Feature contribution was evaluated using Shapley Additive xPlanations across regions [45]. Kit response predictors were identified by performing log-odds ratio of feature occupancies at KREs compared to non-interaction *cis*-elements. Model performance was evaluated by area under receiver operation curve (AUROC) and area under precision-recall (AUPR) curves. Model fitting was checked by k-fold cross-validation

### Scoring

An activity score for each KRE was calculated by adding together the chromatin occupancy of features positively associated with activity prediction, the chromatin accessibility in Kit^+^ cell types, the ABC score, and the presence of acetylated H3K27. Kit expression decreases as EPCs mature, so KREs were given a +2 score if accessible in early erythroblasts (CFUEs, ProEs) and –1 score for accessibility at later stages (BasoE, PolyE, OrthoE) where Kit is downregulated [46]. Kit response predictive features found at KREs were added to the KRE activity score based on the log_e_ (odds-ratio) of its importance. Active elements annotated by ABC scores were given +1 and inactive regions were given –1. H3K27Ac occupancy was +1 and lack of occupancy was -1. KRE scores overall ranged from –4 to +6.15, with the higher numbers predicted to be active Kit-responsive enhancers. PhyloP scores >1 was +1, and the scores were used to annotate for evolutionary conservation status [47].

### Data processing and visualizations

ATAC-seq analysis including the heatmaps and genome wide annotation of peaks were visualized using R Bioconductor libraries – ComplexHeatmap and ChIPseeker [48]. XGBoost analysis was performed using the scikit-learn interface [49] with data processing by pandas and numpy libraries [50]. Model training and evaluation on curated Kit sensitive datasets, and visualization were carried out using seaborn, matplotlib and pandas libraries [51, 52]. Genome tracks were visualized using Integrated Genomics Viewer (IGV) [53] and Venn Diagrams were generated using the web interface – eulerr.co [54].

### Quantitative PCR

Total RNA was isolated from 1 × 10^6^ cells using TRIzol. RNA purity was determined using NanoDrop spectrophotometer (Thermo Scientific). One microgram of RNA was treated with DNaseI and mixed with 200 ng oligo(dT) and 50 ng random hexamer primers prior to reverse transcribing to synthesize complementary DNA (cDNA). qPCR was performed using PowerSYBR Green PCR Master Mix (Applied Biosystems) and 200 nM concentration of site-specific primers, and run on a QuantStudio3 Real-Time PCR System. Relative gene expression was calculated using standard-curve-based relative quantitation, generated from serial dilution of control cDNA. Expression levels were normalized to 18S ribosomal RNA. Control reactions lacking RT were included to verify the absence of genomic DNA contamination, except in cases where DNA was the intended template.

### Statistical analysis

RNA-seq data were statistically evaluated for transcriptional changes using DESeq2 [39]. Statistical analysis for quantitative RT-PCR and Annexin V stained cells were performed using GraphPad Prism version 8. Differential ATAC-seq footprints were identified with TOBIAS, in which subsampling-based empirical *P*-values were computed and adjusted for false discovery using the Benjamini–Hochberg method. Motif enrichment was assessed with HOMER using a hypergeometric test followed by Benjamini–Hochberg correction [33]. Differential chromatin accessibility by MAnorm was tested for significant peaks by using a Bayesian model [55]. XGBoost-predicted features were tested against a null distribution of feature importance scores. Differences in KRE scores across multiple occupancy groups were evaluated using Kruskal–Wallis test, followed by Dunn’s multiple comparison test.

## Results

### The Kit signaling pathway converges on chromatin at AP-1 and EGR motifs

**Figure 1.**
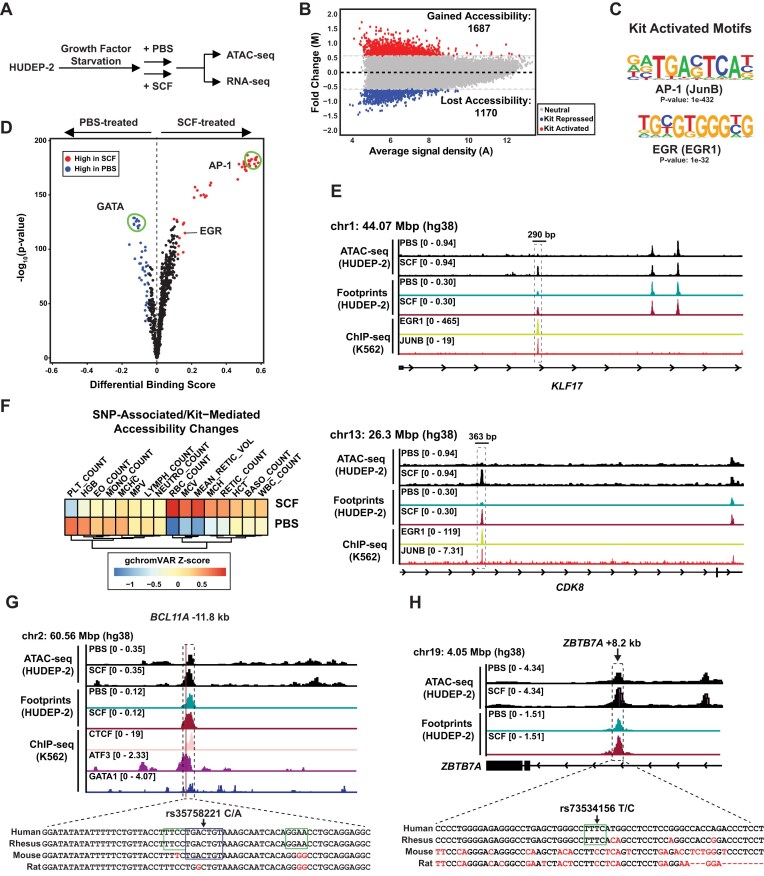
Increased utilization of AP-1 and EGR1 chromatin sites as a response to Kit activation. (**A**) Experimental layout. Growth factor-deprived HUDEP-2 cells were treated with SCF (50 ng/mL) or PBS for 1 h. Cells in both conditions were isolated for ATAC-seq and RNA-seq. (**B**) MAnorm plot graphing signal fold change in response to SCF versus the average signal density (*N* = 2). (**C**) Motif enrichment of AP-1 (JunB) and EGR1 motifs in Kit-activated regions. Motif data from K562 (GSE105243, GSE96382). (**D**) TOBIAS footprinting analysis comparing PBS-treated versus SCF-treated HUDEP-2 cells. (**E**) KLF17 and CDK8 with ATAC-seq (SCF and PBS), TOBIAS footprinting (SCF and PBS), chromatin occupancy of JUNB (AP-1), EGR1 at the KLF17 and CDK8 gene loci (ENCSR7951YP, ENCSR211LTF). (**F**) gChromVAR analysis of regions where accessibility changed between SCF and PBS conditions. (**G**) The rs35758221 SNP near BCL11A is associated with RBC counts. ATAC-seq (SCF and PBS) and TOBIAS footprinting in HUDEP-2, ChIP-seq of CTCF, ATF3, and GATA1 in K562 cells. Boxes indicate sequence with partial AP-1 motif and ETS motifs. (**H**) The rs73534156 SNP in the ZBTB7A intron is associated with RBC counts. ATAC-seq (SCF and PBS) and TOBIAS footprinting in HUDEP-2. Box indicates ETS motif.

Dynamic changes to chromatin accessibility induced by Kit pathway activation are expected to occur at *cis*-elements controlling Kit-dependent cellular processes, including proliferation and differentiation. We evaluated Kit-induced chromatin changes in an erythroid precursor cell lineage dependent on the Kit signaling pathway (HUDEP-2) for survival and proliferation [56]. To map acute, genome-wide chromatin responses to Kit RTK signaling, we grew HUDEP-2 cells in growth factor-deprived media for 5 h and then stimulated the Kit receptor with its ligand, SCF, for 1 h, followed by ATAC-seq to quantify accessibility changes by ATAC-seq (Fig. 1A). The acute approach to stimulate cells with SCF after serum starvation was previously established in primary erythroid precursor cells to evaluate intermediate phospho-proteins involved in acute Kit signaling responses [24, 57, 58].

SCF stimulated rapid and widespread chromatin remodeling, with accessibility increasing at 1687 sites (Kit-activated) and decreasing at 1170 sites (Kit-repressed) (fold-change cut off: 1.5) (Fig. 1B and Supplementary Table S3). We collectively refer to all sites exhibiting measurable Kit-dependent accessibility changes as “Kit response elements (KREs)”. Although unified by shared responsiveness to Kit signaling, KREs exhibited considerable genomic and functional diversity. While 15% of the Kit-activated sites were found at promoter regions, most sites with increased accessibility were in intronic (49%) or distal intergenic (24%) regions, highlighting potential regulatory roles at short and long range (Supplementary Fig. S1A). Motif enrichment analysis revealed an overrepresentation of the AP-1 consensus motif (TGA[N]TCA) and an EGR-like motif (GCGTGGG) across all activated KREs, suggesting that AP-1 and EGR proteins mediate Kit-dependent transcriptional responses in erythroid cells [4] (Fig. 1C and Supplementary Table S4). Among all KREs, AP1 and EGR1 were co-occupied by ChIP in K562 erythroleukemia cells at 526 sites (14%) (Supplementary Fig. S1B and C). To assess Kit-induced changes in protein occupancy at these sites, we applied TOBIAS “footprinting” to ATAC-seq data [34]. Comparisons of TOBIAS footprints across conditions revealed that Kit receptor stimulation increased footprints at AP-1 and EGR motifs, accompanied by a depletion of GATA factor footprints (Fig. 1D and Supplementary Table S5). AP-1 and EGR footprints increased at 984 and 397 KREs, respectively, while GATA motif footprints decreased at 487 sites after Kit activation (Supplementary Table S5). A substantial fraction of AP-1 (40%), EGR (21%), and GATA (42%) footprints occurred within gene introns (Supplementary Fig. S1D), underscoring the importance of intronic *cis*-elements in Kit signaling responses. For example, an intronic Kit-activated *cis*-element in the Krüppel-like transcription factor 17 (KLF17) gene, which regulates the erythroid cell cycle [59], was EGR1- and JUNB-occupied in K562 cells (Fig. 1E). Similarly, a Kit-responsive intronic element within Cyclin Dependent Kinase 8 (CDK8), a known c-Jun co-factor and therapeutic target in acute myeloid leukemia, is EGR1- and JUNB-occupied in published datasets (Fig. 1E) [60].

To assess the possible relevance of KREs to human erythroid phenotypes, we integrated the KRE list with genome-wide association study data using gchromVAR [35]. Kit-responsive chromatin regions were significantly enriched for erythroid trait-associated SNPs (Fig. 1F and Supplementary Table S6). Notably, a red blood cell trait-associated C/A variant (rs35758221) located 11.8 kb upstream of the BCL11A transcription start site overlapped with a KRE and a weak AP-1 motif at the same location (Fig. 1G). BCL11A is a central regulator of the transcriptional switch from fetal to adult hemoglobin expression important for post-natal red blood cell functions [61, 62]. The BCL11A −11.8 kb KRE also contains GATA and ETS motifs, and is occupied by the AP-1 family member ATF3 in K562 cells (Fig. 1G). Elsewhere, a T/C variant (rs73534156) in an intron of the ZBTB7A gene (ZBTB7A + 8.2 kb) determines whether this KRE has an intact ETS factor binding motif and is associated with red blood cell traits, suggesting that the variant may impact transcription factor binding and *cis*-element function (Fig. 1H). ZBTB7A regulates erythroid progenitor apoptosis by controlling B-cell lymphoma 2-interacting mediator of cell death (*BIM*) and fetal hemoglobin expression [63, 64]. Overall, these data define the genome-wide landscape of erythroid KREs, revealing common transcription factor occupancy patterns downstream of growth factor signaling and linking Kit-dependent chromatin remodeling to human erythroid trait-linked genetic variation.

**Figure 2.**
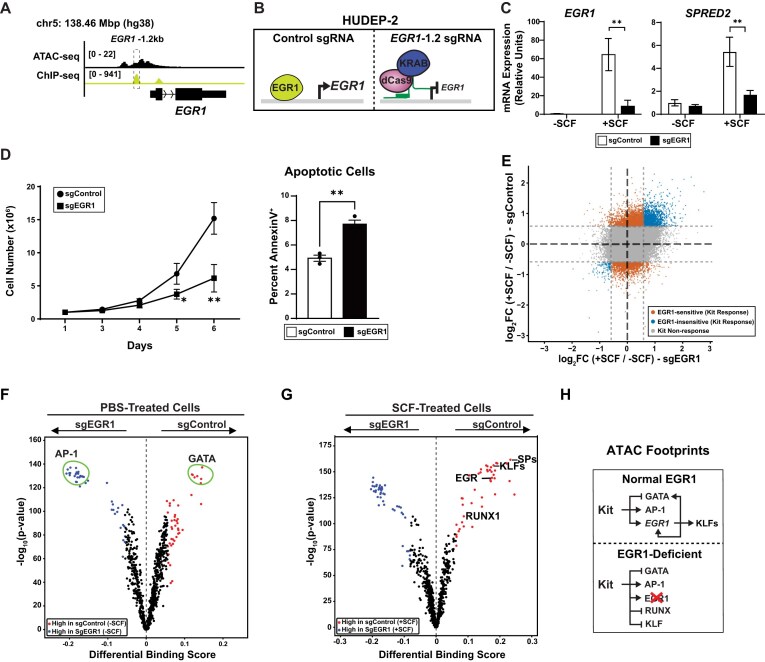
EGR1 deficiency drives distinct Kit responses. (**A**) EGR1-KRE (sgEGR1) identified −1.2 kb from EGR1 transcription start site. (**B**) Experimental design to disrupt the EGR1-KRE using CRISPRi. (**C**) qPCR quantitation of EGR1 and SPRED2 mRNA levels in HUDEP-2 cells (*N* = 3). (**D**) (left) Expansion rate of HUDEP-2 cells following lentiviral transduction with sgEGR1 or controls. (Right) Flow cytometric analysis of the percentage of AnnexinV+ HUDEP-2 cells after 5- days in culture (*N* = 3). Error bars represent standard deviation (SD). *P < .05; **P < .01; ***P < .001 (two-tailed unpaired Student’s *t*-test). (**E**) Heatmap depicting changes to ATAC-seq signal in sgEGR1 HUDEP-2 cells following Kit receptor stimulation. Dotted lines indicate fold-change cutoff of 1.5. (**F**) TOBIAS footprint comparison of control cells expressing sgEGR1 or sgScrambled controls. (**G**) TOBIAS footprint comparison of SCF-treated cells expressing sgEGR1 or sgScrambled controls. (**H**) Working model developed from ATAC footprint data representing motifs used by Kit response pathways in the presence or absence of EGR1.

EGR family transcription factors function broadly across hematopoietic, neural, and cancer contexts [65–67]. In our analysis, EGR1 emerged as a gene that is strongly Kit-responsive, the EGR1 locus contains a KRE, and EGR1 binding motifs were increased at other KREs genome-wide after Kit activation (Fig. 2A). These findings suggested that EGR1 may act as a key effector of Kit-dependent chromatin changes. To test whether EGR1 is needed for Kit-induced accessibility changes, we targeted a −1.2 kb KRE at the EGR1 locus using dCas9-KRAB-mediated CRISPR interference (CRISPRi) (Fig. 2B). Targeting this element decreased Kit-induced EGR1 expression, reducing *EGR1* messenger RNA (mRNA) by 7.5-fold versus sgControl cells (*P* = .006) (Fig. 2C). Consistent with impaired EGR1 activity, expression of Sprouty-related EVH1 domain protein 2 (*SPRED2*), a Kit and EGR1 target gene, was 3.2-fold lower following Kit activation in sg*EGR1*-infected cells compared to sgControl cells (*P* = .008) (Fig. 2C). Functionally, we tested whether disruption of the EGR1 KRE altered cell growth or survival. Cells expressing sg*EGR1* grew 2.5-fold (*P* = .001) slower and cultures contained 1.6-fold more apoptotic cells (Annexin V^+^) relative to control cells (*P* = .002) (Fig. 2D and Supplementary Fig. S2A), consistent with a role for EGR1 in supporting Kit-driven erythroid precursor cell growth and survival.

Next, we asked whether Kit-induced chromatin accessibility changes depend on EGR1 upregulation. ATAC-seq signal was quantified across all four conditions, and peaks were classified based on their Kit responsiveness in sgControl- versus sg*EGR1*-expressing cells (Supplementary Fig. S2B). Among 3812 analyzed coordinates, 2371 (62.2%) Kit-responsive peaks were EGR1-sensitive (FC > 1.5, *P* < .05) whereas 1441 were EGR1-insensitive (Fig. 2E and Supplementary Table S7). These results indicate that EGR1 functions downstream of Kit signaling to enable chromatin opening. Consistent with this, sg*EGR1*-expressing cells exhibited altered *cis-*element usage genome-wide. Specifically, sg*EGR1*-expressing cells used GATA sites less frequently and AP-1 sites more frequently than control cells (Fig. 2F and Supplementary Table S8). Following acute Kit activation, we saw selective use of EGR1 motifs in control cells versus sgEGR1-expressing cells (Fig. 2G) (differential binding score +0.2). Other EGR1-sensitive, Kit-activated footprints were consensus binding motifs for transcription factors and chromatin remodelers, including KLFs, SPs, CTCF, and RUNX (Fig. 2G). The same sequence motifs were enriched at EGR1-sensitive KREs versus EGR1-insensitive KREs based on reference genome sequence (Supplementary Fig. S2C). Using changes to ATAC footprinting induced by Kit activation, we can model distinct motifs govern EGR1-sensitive versus EGR1-insensitive chromatin responses to Kit signaling. Disruption of EGR1-dependent KRE activation rewires *cis*-regulatory logic, shifting the erythroid precursor cells away from GATA-centered regulatory programs toward a greater reliance on AP-1 motif usage (Fig. 2H).

### Chromatin features at KREs can predict Kit transcriptional activation

**Figure 3.**
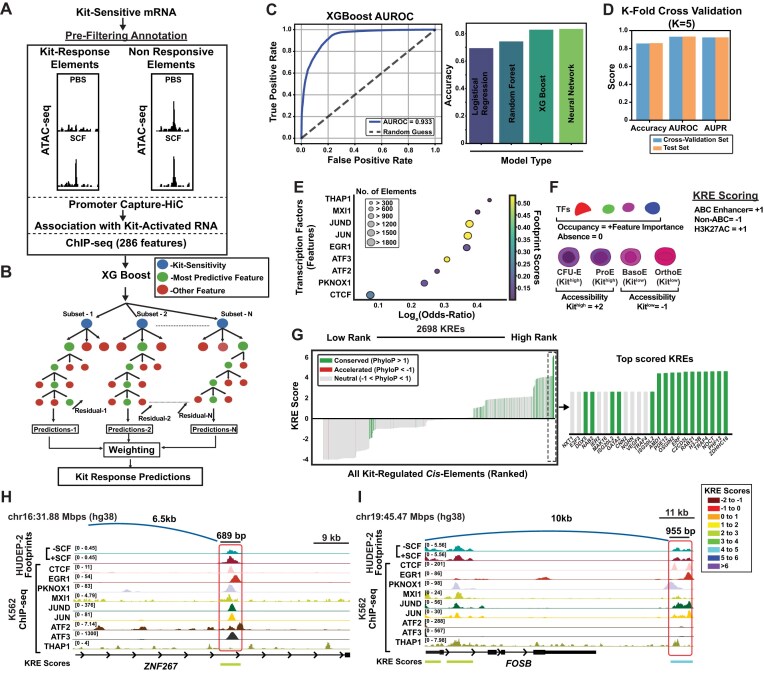
Kit transcriptional activation is predictable based on chromatin-feature machine learning. (**A**) To build a prediction model for *cis-*element control of Kit signaling, KREs were annotated to nearby and interacting Kit-regulated mRNAs based on accessibility, PC-HiC, and chromatin occupancy of 286 sequence features. (**B**) Schematic representation of XGBoost model using iterative learning to identify chromatin features associated with either Kit-sensitive or Kit-insensitive KRE-associated transcripts. (**C**) AUROC scores from experimental model related to true versus false positives is plotted against random guesses (*K* = 5). (**D**) *K*-fold cross validation scores for model accuracy, AUROC, and AUPR. XGBoost compared to other learning methods (logistic regression, random forest, and neural networks). (**E**) Kit-response predictive features were ranked by log(odds ratio) values, motif footprint presence with SCF, and occupied in at least 10% of KREs. (**F**) Scoring for prioritized KREs utilized a scoring system for occupancy of predicted features, accessibility in relevant cell types, and enhancer activity. (**G**) Score distribution across the KREs mapped with higher scores indicative of enhancer activity for that KRE, colored by PhyloP evolutionary conservation. Inset provides annotation of top-ranked loci (duplicate target transcripts were removed). (**H**) Representative example of KRE accessibility and occupancy at the ZNF267 locus. (**I**) Representative example of KRE accessibility and occupancy at the FOSB locus. ATAC-seq (±SCF) (GSE314033) and ENCSR211LTF (EGR1), ENCSR115SMW (PKNOX1), ENCSR000EGZ (MXI1), ENCSR000EGN (JUND), ENCSR000EFS (JUN), ENCSR869IUD (ATF2), ENCSR028UIU (ATF3), ENCSR000BNN (THAP1), and ENCSR000EGM (CTCF). Genomic coordinates are shown at the top left of each panel.

Next, we constructed a molecular profile for each KRE to allow functional prediction of Kit-responsive *cis*-regulatory activity (Supplementary Fig. S3). Since KREs are expected to control transcription through both short- and long-range interactions with target promoters, we annotated KREs to genes using two strategies: nearest-gene (<15 kb) and PC-HiC interactions derived from primary human erythroblasts [40]. To define the molecular features associated with each KRE, we integrated transcription factor occupancy data from K562, HUDEP-2, and primary erythroblast cells using publicly available datasets (ENCODE, ChIP-Atlas) [42, 43] (Supplementary Table S9). After quality filtering, 286 of the 376 total transcription factors were considered as features. We then linked these annotated *cis*-elements to Kit-regulated transcriptional outputs by integrating RNA-seq data from HUDEP-2 cells collected at the same time point post SCF stimulation. We identified 375 Kit-activated and 138 Kit-repressed transcripts were linked to 2698 accessible *cis*-elements (including both KREs and Kit-insensitive regions) (FDR ≤ 0.05) (Fig. 3A). In human erythroid precursors derived from CD34^+^ cells, 33.8% of the Kit-activated transcripts were shared with Kit-activated transcripts in HUDEP-2, supporting the premise that shared and distinct Kit-dependent transcriptional programs exist between HUDEP-2 and primary cells (Supplementary Fig. S4A and Supplementary Table S10). [40, 68].

Using this information, we trained a machine learning model using XGBoost classifiers [44] (Fig. 3B). The goal in building this model was to better understand the relationships between multiple factors and their roles in influencing signaling outcomes. XGBoost is optimal for training this model since the weighted decision tree yields an interpretable output, which allows for assigning specific high-weight features to individual KREs. The chromatin-accessible regions which were linked to Kit-insensitive transcripts served as a negative-control dataset for training the model. The model was 86% accurate at predicting Kit responsiveness, with an AUROC score of 0.93, and accuracy of XGBoost was comparable with other published classifier tools (Fig. 3C). Additional performance metrics, including accuracy, AUROC, and AUPR suggested good model performance without overfitting (Fig. 3D, Supplementary Fig. S4B, and Supplementary Table S11). An analysis of the most important chromatin features weighted by the decision tree revealed that high predictive power was driven by transcription factors associated with inflammatory and immediate early responses (JUN, JUND, ATF2, ATF3, and EGR1) [8, 69, 70] and chromatin architectural regulators (CTCF) [71, 72] (Fig. 3E), largely consistent with ATAC footprinting data. These results demonstrate that integrated molecular annotation of KREs enabled accurate prediction of *cis*-regulatory responses to Kit signaling. Our approach captured shared regulatory logic across hundreds of *cis*-elements to reveal a core set of transcriptional and chromatin-based predictors which can be used to derive mechanisms of Kit-mediated control of erythroid gene expression.

To stratify *cis*-elements by their predicted functional roles in Kit signaling, we assigned an integrated activity score to each KRE. The KRE score used three complementary criteria: (i) transcription factor occupancy weighted by model-derived feature importance scores (Supplementary Fig. 4C and D; Supplementary Table S9); (ii) enhancer activity potential, quantified by H3K27ac histone marks and the ABC scores derived from RNA FlowFISH-validated enhancer-promoter interactions in K562 cells [41]; and (iii) chromatin accessibility across relevant Kit-expressing primary hematopoietic cell types, including dynamic accessibility changes in response to Kit signaling (Fig. 3F) (Supplementary Table S9). Across all KREs, scores ranged between −4 and 6.15, with higher scores predicting a greater *cis*-element activation in response to Kit signaling and lower scores indicating inactivity. To annotate evolutionarily preserved sequences, which may correlate with functional importance, we mapped KREs to PhyloP scores across 100 vertebrate species [73]. Among the highest-scoring KREs (score ≥ 4.0), 29% (45 of 157) exhibited evolutionary conservation (Phylo*P* > 1), indicating selective pressure on signal responsive regulatory elements (Fig. 3G). Since footprinting data showed higher EGR and AP-1 motif utilization after Kit activation, and frequent KREs which were co-occupied by both factors (Supplementary Fig. S4D), we evaluated whether occupancy of these factors was associated with high- or low-scored KREs. Not surprisingly, higher predicted KRE scores were found at KREs with occupancy of EGR1 and/or AP-1 transcription factors, and average baseline scores for KREs lacking both factors was below 0 (Supplementary Fig. S4E and F; Supplementary Table S12). Thus, we validated that XGBoost prediction modeling matches with empirical data from Kit-activated ATAC-seq changes, and suggest that EGR and AP-1 motifs may cooperatively drive KRE activity.

Notably, the top-ranked KREs were associated not only with established Kit target genes but also with several poorly characterized or unannotated loci. The highest-scored KREs were present at canonical hematopoietic genes, including *GATA2*, vascular endothelial growth factor A, and *E2F3*. While several of these genes are known to function downstream of Kit signaling in hematopoiesis [74–76], *GATA2* has not been previously described as a Kit target gene. KREs predicted to drive pathway-dependent transcriptional activation were linked to transcription start sites of empirically-validated Kit-regulated transcripts. For example, a KRE associated with zinc finger protein 267 (*ZNF267*) was located +6.5 kb upstream of the *ZNF267* transcription start site (Fig. 3H), and a FOSB KRE was located in a region 10 kb downstream of the *FOSB* promoter (Fig. 3I). Collectively, these analyses demonstrate that integration of chromatin occupancy and accessibility signatures at promoter-annotated ATAC-seq data can accurately predict which genes may respond to signal-responsive chromatin changes, yielding a framework for interpreting Kit-dependent *cis*-regulatory logic.

**Figure 4.**
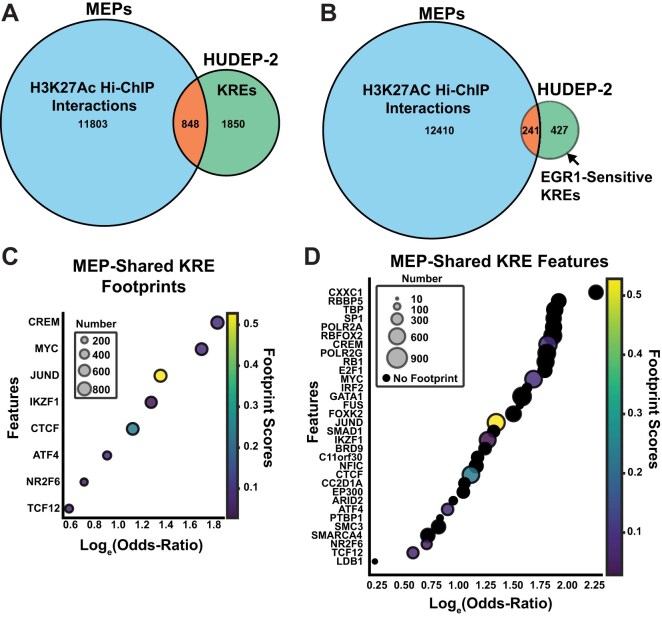
Predicting Kit response activities in MEPs. (**A**) Venn diagram comparing genomic coordinates of all KREs mapped in HUDEP-2 cells to the list of H3K27Ac Hi-ChIP interactions. (**B**) Venn diagram comparing genomic coordinates of EGR1-sensitive KREs mapped in HUDEP-2 cells to the list of H3K27Ac Hi-ChIP interactions. (**C**) Predictive features in shared MEP-HUDEP-2 KREs containing footprint scores ranked by log (odds ratio) values. Size of dots indicate the number of KREs that contain that feature. (**D**) Predictive features with and without footprint data ranked by log (odds ratio) values. Size of dots indicate the number of KREs that contain that feature.

Next, we tested whether our model could predict dynamic signal-responsive enhancers operating in other cell types. Primary human megakaryocyte-erythrocyte progenitors (MEPs) are Kit-positive and capable of differentiation into proerythroblasts [77]. Also, MEP promoter-enhancer interactions have been previously mapped by H3K27Ac HiChIP [78, 79]. We used these data to map overlapping accessible *cis*-elements in MEPs and HUDEP-2. Overall, 53.6% (1448/2698) of HUDEP-2 KREs were chromatin accessible in MEPs. Among the HUDEP-2 KREs we previously annotated, 31.4% (848/2698) were annotated in MEPs as regulatory enhancers based on H3K27Ac HiChIP (Fig. 4A), suggesting that MEPs and HUDEP-2 share chromatin landscapes which may permit Kit activity predictions. A similar percentage (28.4%) of the KRE subgroup which were EGR1-sensitive corresponded with annotations of active enhancers in MEPs (Fig. 4B). We followed the workflow from Fig. 3A to evaluate Kit responsiveness at overlapping KREs, using the HUDEP-2 uniquely accessible KREs as a control dataset. While empirical validation is lacking, the output of this analysis could predict Kit targe gene activation with 89% accuracy (Supplementary Fig. S5A and Supplementary Table S13). Among the list of shared KREs with measurable ATAC footprint scores, the model listed several predictive features that overlapped with the broader HUDEP-2 list (e.g. JUND, CTCF, and ATF4) (Fig. 4C). Our model also identified additional transcription factor features that uniquely distinguish shared KREs compared to those unique to HUDEP-2 (e.g. GATA1, MYC, NR2F6) (Fig. 4D). 11% (94 of 848) of shared KREs were among the highest scored (score ≥ 4.0), and 8% (68 of 848) of shared KREs were evolutionarily conserved (Supplementary Fig. S5B). While the predictions have not yet been functionally tested in MEPs, our analyses suggest that predictions of Kit response activities can identify candidate *cis*-elements that coordinate chromatin and transcriptional outputs across multiple cell types.

### EGR1-sensitive KREs mediate Kit-dependent transcription

Since Kit-dependent *cis*-regulatory responses can be predicted using gene expression and occupancy data, we next asked whether EGR1-dependent Kit responses are similarly predictable. RNA-seq analysis of SCF-stimulated HUDEP-2 cells expressing sg*EGR1* or sgScrambled found 127 Kit-regulated transcripts were EGR1-sensitive, while 326 were EGR1-insensitive (Supplementary Table S14). The EGR1-sensitive transcripts were associated with 2070 KREs, defining a large subset of *cis*-elements whose activity depends on EGR1 upregulation (Supplementary Table S14).

**Figure 5.**
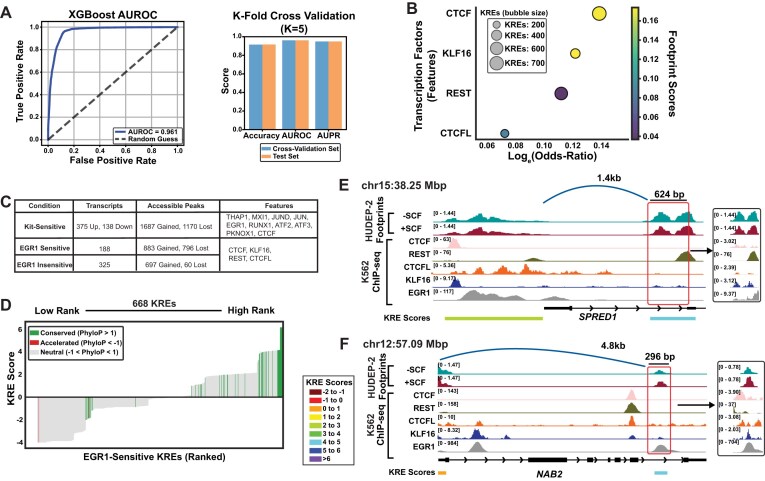
EGR1-sensitive targets of Kit signaling have a distinct molecular profile. (**A**) AUROC scores using KREs associated with EGR1-sensitive and -insensitive mRNA transcripts and *K*-fold cross validation (*K* = 5). (**B**) EGR1-sensitive features were identified based on log (odds ratio) and motif footprint scores. (**C**) Table of Kit-sensitive and EGR1 sensitive features at KREs. (**D**) Ranked list of KRE scores in EGR1-sensitive KREs. (**E**) Representative example of EGR1-sensitive KRE accessibility and occupancy at the SPRED1 locus. (**F**) Representative example of EGR1-senstivie KRE accessibility and occupancy at the NAB2 locus. ATAC-seq (± SCF) (GSE314033) and ChIP-seq [ENCSR211LTF (EGR1), ENCSR137ZMQ (REST), ENCSR760UVO (KLF16), ENCSR000EGM (CTCF), and ENCSR000BNK (CTCFL)] with PhyloP and KRE scores. Genomic coordinates are shown at the top left of each panel.

We trained a machine learning model based on the RNA-seq data to distinguish EGR1-sensitive KREs from a matched set of EGR1-insensitive KREs. The model achieved 92% accuracy predicting whether EGR1 upregulation was needed for Kit-dependent gene activation (AUROC = 0.96) (Fig. 5A), indicating that a signature of EGR1 dependency is encoded in its *cis*-regulatory features. Feature importance analysis identified 20 predictors of EGR1 sensitivity, four of which (REST, KLF16, CTCF, and CTCFL) were present at >10% of KREs (Fig. 5B). EGR1-sensitive and -insensitive predictive features include transcription factors which overlap with Kit-sensitive features, and unique features which may indicate mechanistic differences (Fig. 5C). Interestingly, EGR1 was ranked low on the list of important features predicting EGR1-sensitive Kit activation. We attribute this finding to the occupancy of EGR1 at many KREs which were insensitive to EGR1 levels after Kit activation. Notably, REST has established regulatory roles in hematopoiesis [80], KLF16 functions as a transcriptional repressor [81], and CTCF and CTCFL are important chromatin architectural proteins [71, 82]. Across the complete set of the 2698 KREs linked to Kit-responsive genes, 668 were classified as EGR1-sensitive. The highest-scored EGR1-sensitive KREs were evolutionarily conserved and associated with genes including *NAB2, FOSL2*, and *SPRED1* (Fig. 5D and Supplementary Table S15). Our model predicts that the *SPRED1* KRE located +1.4kb from its TSS, directly regulates *SPRED1* expression in an EGR1-dependent manner (Fig. 5E). Although *NAB2* is known to modulate *EGR1* activity, its role in erythropoiesis is poorly defined [83]. In our data, both *NAB2* and *EGR1* were upregulated by Kit activation. The presence of an EGR1 sensitive KRE located +4.8 kb from the *NAB2* promoter suggests a potential feed forward loop in erythroid precursors (Fig. 5F). We trained our model on this group of KREs using EGR1-insensitive KREs as a control group (Supplementary Fig. S5C). In this analysis, five features were found to predict EGR1-sensitive Kit activity in MEPs, including transcription factors KLF1, SP1, and ETV6 (Supplementary Fig. S5D and E; Supplementary Table S16), and captured most of the evolutionarily conserved and highly ranked KREs (Supplementary Fig. S5F and Supplementary Table S16). These results demonstrate that chromatin features at KREs can accurately predict EGR1 dependency within the broader framework of Kit signaling-dependent chromatin changes, revealing *cis*-regulatory logic that governs chromatin responses to growth factor stimulation. Our analysis predicts that distinct transcriptional complexes may be involved in driving Kit-dependent gene regulation at *cis*-elements which are commonly accessible between progenitor cell types and HUDEP-2, compared to *cis-*elements uniquely accessible in HUDEP-2 cells.

**Figure 6.**
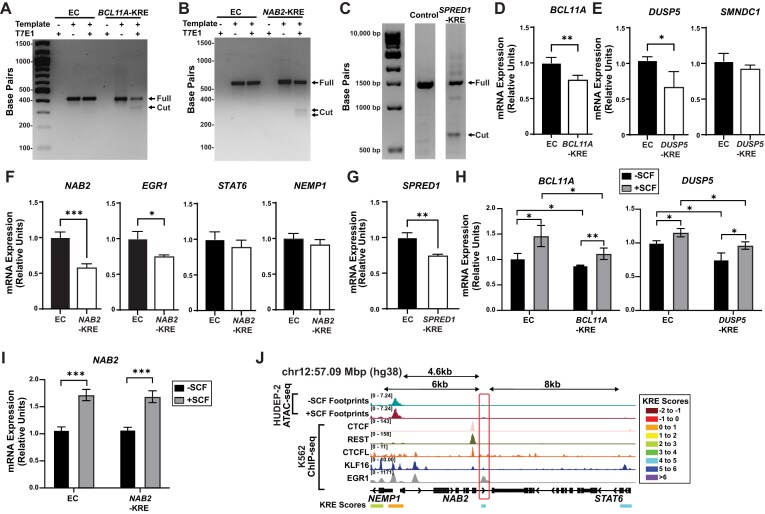
Targeted deletions of KRE sequences impact target gene expression levels. (**A**) Agarose gel electrophoresis of a PCR-amplified region surrounding the *BCL11A*-KRE (−11.8 kb) using DNA template isolated from control-infected (EC) and *BCL11A*-KRE-targeted cells and treated with the T7 endonuclease I. Cut band represents gene-edited DNA. (**B**) Agarose gel electrophoresis of a PCR-amplified region surrounding the *NAB2*-KRE (+4.8 kb) using DNA template isolated from control-infected (EC) and *NAB2*-KRE-targeted cells and treated with the T7 endonuclease I. Cut band represents gene-edited DNA. (**C**) Agarose gel from a PCR amplified region surrounding the *SPRED1*-KRE (+1.4 kb) using DNA template isolated from control-infected (EC) and *SPRED1*-KRE-targeted cells and treated with the T7 endonuclease I. Cut band represents gene edited DNA. (**D**) Quantitative real-time PCR of *BCL11A* mRNA expression from control (EC) and gene-targeted (*BCL11A*-KRE) cells (*N* = 3). (**E**) Quantitative real-time PCR of *DUSP5* and *SMNDC1* mRNA expression from control (EC) and gene-targeted (*DUSP5*-KRE) cells (*N* = 3). (**F**) Quantitative real-time PCR of *NAB2, EGR1, STAT6*, and *NEMP1* mRNA expression from control (EC) and gene targeted (NAB2-KRE) cells (*N* = 3). (**G**) Quantitative real-time PCR of SPRED1 mRNA expression from control (EC) and gene targeted (*SPRED1*-KRE) cells (*N* = 3). (**H**) Quantitative real-time PCR of BCL11A and DUSP5 mRNA expression in control (EC) and gene-targeted (*BCL11A*-KRE, *DUSP5*-KRE) cells before or after SCF stimulation (*N* = 3). (**I**) Quantitative real-time PCR of *NAB2* mRNA expression in control (EC) and gene-targeted (*NAB2*-KRE) cells before or after SCF stimulation (*N* = 3). Error bars represent SD. *P < .05; **P < .01; ***P < .001 (two-tailed unpaired Student’s *t*-test). (**J**) KRE accessibility and occupancy at the *NAB2* locus annotated with predicted nearby *NAB2*-KREs and distances to the transcription start site. ATAC-seq (±SCF) (GSE314033) and ChIP-seq [ENCSR211LTF (EGR1), ENCSR137ZMQ (REST), ENCSR760UVO (KLF16), ENCSR000EGM (CTCF), and ENCSR000BNK (CTCFL)] with KRE scores. Genomic coordinates are indicated in the top left.

To test *cis*-element activities predicted by our model, we selected several of the high-scoring Kit-sensitive and EGR1-sensitive KREs for targeted genetic deletion in HUDEP-2 cells. Deletion efficiency was evaluated by T7 endonuclease digestion following PCR amplification of the genomic region surrounding the EGR1-insensitive *BCL11A* gene (Fig. 6A) and *NAB2* (Fig. 6B) KREs. Because the *SPRED1* and *DUSP5* KREs spanned larger regions of DNA, we used two sgRNAs to delete the entire elementand confirmed deletion by gel electrophoresis and Sanger sequencing (Fig. 6C and Supplementary Fig. S6A and B). Consistent with model predictions, KREs from both Kit-sensitive and EGR1-sensitive groups contained transcription factor binding motifs associated with inflammatory and early response pathways (Supplementary Fig. S6C). Disruption of each element led to specific and quantitative changes in target gene expression. Deletion of the −11.8 kb *BCL11A* KRE resulted in a 1.3-fold reduction in *BCL11A* mRNA relative to control cells (*P* = .003) (Fig. 6D). Similarly, removal of the distal −221 kb *DUSP5* KRE reduced *DUSP5* mRNA expression by 1.6-fold (*P* = .042), while expression of a nearby gene (*SMNDC1*) remained unchanged, indicating regulatory specificity (Fig. 6E). It is worth noting that we used nucleofected populations of edited and unedited cells to test gene expression changes due to concern that extensive time required in culture for single cell cloning may impact cell phenotypes, so it is possible that these results underestimate the impact of each KRE on gene expression.

We next examined two high scoring EGR1-sensitive candidates predicted to control *NAB2* and *SPRED1* mRNA expression (KRE score > 4). Deletion of the +4.8 kb *NAB2* KRE led to a 1.4-fold decrease in *NAB2* expression compared to non-targeting controls (*P* = .001). Notably, *EGR1* expression was also reduced by 1.3-fold following *NAB2*-KRE deletion (*P* = .014), suggesting a previously unrecognized regulatory relationship between *NAB2* and *EGR1* in erythroid cells (*P* = .014). Expression of nearby genes (*STAT6* and *NEMP1*) were not affected by *NAB2* KRE gene editing (Fig. 6F). Although NAB2 has been described as a transcriptional corepressor in other contexts [83], these findings indicate that NAB2 promotes *EGR1* expression in erythroid precursors via a Kit response *cis*-element predicted by our model. Finally, deletion of the extended *SPRED1* KRE using paired sgRNAs resulted in a 1.3-fold reduction in SPRED1 expression relative to controls (*P* = 0.003), validating its role as a functional Kit-responsive regulatory element (Fig. 56). Next, gene edited cells were serum starved and SCF-stimulated to test whether Kit-mediated upregulation required the KRE. While knockout cells were SCF responsive, deletion of the KREs interacting with *DUSP5* (DUSP5 -221kb) and *BCL11A* (BCL11A -11kb) significantly attenuated the response at the mRNA level (Fig. 6H). Deletion of the NAB2 KRE, while sufficient to lower gene expression in full media, did not affect the Kit-mediated NAB2 upregulation (Fig. 6I). We suspect this is due to the presence of two additional KREs at the same locus which may functionally compensate during acute Kit stimulation (Fig. 6J). Taken together, genetic deletion of KREs confirm their direct control of target gene expression, many of which are required for fine-tuning Kit signaling in erythroid precursors. Importantly, these results demonstrate that our model accurately predicts dynamic *cis*-element regulatory function with transcriptomics data, providing a generalizable framework for decoding signal-dependent gene regulation.

## Discussion

Computational approaches can predict *cis*-regulatory element activity under steady-state conditions, using chromatin state annotations, 3D genome organization, and accessibility features to define promoters and enhancers genome-wide [10, 13, 16, 84]. Complementary experimental strategies, including the ABC model, improve functional inference by integrating predictions with high-throughput validation, enabling accurate identification in defined cell contexts [41]. However, most assays rely on static measurements and lack environmental context, limiting their ability to predict how *cis*-elements respond to acute signaling inputs. Our model addresses this limitation by explicitly integrating chromatin accessibility dynamics, transcription factor occupancy features, and gene expression outputs following acute activation of Kit signaling. Pathway-responsive transcription factors such as EGR1 and AP-1 can be captured with this approach which are masked in steady state chromatin environment data. Our model achieves high predictive accuracy using transcriptomic data as the experimental input, enabling the inference of dynamic *cis*-element functions. The approach adds mechanistic understanding related to how extracellular signals are decoded at the level of chromatin to drive transcriptional programs.

Although we primarily used this model to investigate Kit receptor activation in erythroid precursors, we also identified a subset of Kit responsive *cis*-elements which share activity in primary human MEPs, and it can theoretically test *cis*-element activities within other growth factor pathways, intersecting pathway inputs, stress responses, or developmental transitions as well. Our approach is agnostic to pathway or cell type, relying instead on mRNA levels and chromatin accessibility, and then inferring transcription factor occupancy and promoter contacts based on published data. In erythropoiesis, it is likely that the KREs we identified have context-dependent functions. For example, some KREs may be Kit-activated only during development, while distinct KREs may act as anemia stress-response elements or may function as required components in other signaling pathways. It is instructive to consider the context of “stress erythropoiesis”, during which sub-populations of erythroid precursor cells with distinct behavioral characteristics emerge in acute anemia to regenerate lost erythrocytes [8, 28, 85, 86]. Since this environment is characterized by rapid cell expansion and differentiation, and *cis*-element activities are dependent on Kit signaling and inflammatory stress cues, our model can be applied to infer (and ultimately test) what distinct regulatory elements are involved in anemia recovery mechanisms. By applying the approach to investigate more complex forms of physiological or pathological stress, we can identify *cis*-elements that control acute stress responses.

In addition to improving our understanding of signal-dependent gene regulation, dysfunction at KREs may contribute to chromatin misregulation in hematologic malignancies, particularly in settings where Kit (or other RTK signaling pathways) are chronically activated or therapeutically inhibited (reviewed in [87]). Most of our predicted KREs and target genes are poorly characterized. In principle, previously unrecognized genetic circuits downstream of signal activation can contribute to disease progression or treatment failure. Enhancer–promoter networks and/or target genes may provide alternative or complementary therapeutic inroads. Moreover, since KREs overlap with hematopoietic trait–associated genetic variants, non-coding variation at signal-responsive *cis*-elements may modulate red blood cell traits, disease susceptibility, and recovery following hematologic stress. This observation aligns with prior studies demonstrating that variation within *cis-*regulatory elements contributes to hematologic phenotypes [61, 88, 89]. Clinical variability in hematologic disease and treatment response is likely to be shaped by the integrity and regulatory function of signal-responsive *cis*-elements.

We established a multi-tiered, supervised learning model to discover and validate signal-responsive *cis*-elements. Our approach addresses some of the limitations in existing *cis*-element annotations, since a single environmental input variable can alter thousands of accessibility sites genome wide. While these findings establish the predictive framework, several limitations still exist. Our use of RNA-seq as a proxy for transcription does not directly measure it and may be influenced by other factors like transcript length, stability, splicing, processing and rates of transcription. Second, although ATAC-seq footprints provide valuable information regarding motif-level accessibility changes, it infers transcription factor occupancy. Some transcription factors are known to come on and off chromatin without making measurable footprints [90]. Integration of additional direct evidence of transcription and occupancy changes associated with specific environmental responses will be necessary to validate factor-specific binding and refine mechanistic models.

## Supplementary Material

gkag505_Supplemental_Files

## Data Availability

RNA-seq raw and processed files were deposited in the Gene Expression Omnibus database under accession numbers GSE314034, GSE314032, and GSE314033. Code is provided on GitHub: https://github.com/rahuldogiparthi/Cis-Element-Activity-Predictor and on Zenodo (DOI: 10.5281/zenodo.18292661). Public ChIP-seq datasets used in this study were obtained from the ENCODE project and ChIP-Atlas (Table). PhyloP conservation scores were obtained from the UCSC Genome Browser (hg38; 100-way vertebrate) [https://hgdownload.cse.ucsc.edu/goldenpath/hg38/phyloP100way/hg38.phyloP100way.bw]. Genome browser visualizations were generated in IGV using our ATAC-seq data (GSE314033) and publicly available ENCODE ChIP-seq datasets. The ENCODE accession numbers used for visualization include ENCSR211LTF (EGR1), ENCSR795IYP (JUNB), ENCSR115SMW (PKNOX1), ENCSR000EGZ (MXI1), ENCSR000EGN (JUND), ENCSR000EFS (JUN), ENCSR869IUD (ATF2), ENCSR028UIU (ATF3), ENCSR000BNN (THAP1), ENCSR137ZMQ (REST), ENCSR760UVO (KLF16), ENCSR000EGM (CTCF), and ENCSR000BNK (CTCFL).
