# Supplementary material for: Predictive modeling of signal-responsive cis-elements in human red blood cell precursors
Source: Nucleic Acids Res. 2026 May 21;54(10):gkag505. doi: 10.1093/nar/gkag505 (PMC13191290; doi:10.1093/nar/gkag505)
Supplement: gkag505_Supplemental_Files [file gkag505_supplemental_files.zip › rd_pap1_Supplement_revised.pdf]

Supplemental Materials

Dogiparthi *et al.*

Predictive Modeling of Signal-Responsive *Cis*-Elements In Human Red Blood Cell Precursors

This Supplement contains:

6 Figures

6 Figure legends

16 Tables

Supplemental Figure legends

Supplementary Fig 1: Chromatin accessibility changes following Kit pathway activation. A) Genomic annotations of the ATAC-seq peak locations where accessibility increased or decreased in response to Kit pathway activation. B) Stacked bar chart indicates the number of KREs which were occupied by AP-1, or EGR1, or both AP-1 and EGR1. C) Upset plot depicts the numbers of AP1 and EGR1 occupied KREs and relative overlap between conditions. D) Genomic annotations of the ATAC-seq peak locations at specific footprint scores (identified by TOBIAS) that increased in response to Kit pathway activation. Sequences mapped in hg38.

Supplementary Fig 2: Apoptosis response induced by CRISPRi-mediated interference with the KRE at the EGR1 locus. A) Representative flow cytometry between sgControl and sg*EGR1* stained with Annexin V antibody and DAPI (N=3). B) Grid-based classification of Kit-induced chromatin accessibility in sgControl and sgEGR1 cells. C) Co-factor motifs enriched at EGR1-Sensitive KREs compared against EGR1-Insensitive KREs.

Supplementary Fig 3. Multi-omics integration approach to evaluate and prioritize KREs. Schematic representation of experimental approach and annotation criteria to identify Kit-regulated cis-elements that may control erythropoiesis.

Supplementary Fig 4: Modeling chromatin responses to Kit signaling across multiple erythropoietic cell types. A) Venn diagrams of RNA-seq data generated from erythroid differentiated $CD34^+$ cells and HUDEP2 cells which were SCF-starved and stimulated with either SCF (50 ng/mL) or PBS for 1 hour. B) Kit sensitive training data evaluated against different classification models by AUROC and AUPR curves in HUDEP-2 cells. C) Ranked importance scores of chromatin features identified by XGBoost. Importance scores were used in prioritization ranking of KREs. D) Stacked bar chart depicts percentages of KREs occupied by AP1, EGR1, or both. E) Box plot of KRE scores among KREs which were occupied by AP-1-only, EGR1-only, neither or both. *$p<0.05$; ***$p<0.001$ (Kruskal-Wallis with Dunn's test) F) Predictors of EGR1 sensitivity observed by Log (Odds-Ratio) of occupancies compared between EGR1-sensitive to EGR1-insensitive KREs.

Supplementary Fig 5: Evaluating model generalizability in megakaryocyte-erythrocyte progenitors. A) AUROC scores of KREs associated with KREs shared between MEP and HUDEP-2 and KREs unique to HUDEP-2. B) Score distribution of shared MEP-HUDEP2 KREs colored by PhyloP evolutionary conservation. C) AUROC scores of EGR1-sensitive KREs shared between MEP and HUDEP2. D) Predictors of EGR1-sensitive KREs shared between MEPs and HUDEP-2 containing footprint scores and listed by Log (Odds-Ratio). E) All predictors of EGR1-sensitive KREs shared between MEPs and HUDEP-2 and listed by Log (Odds-Ratio). F) Score distribution across the EGR1-Sensitive KREs in MEPs mapped with higher scores indicative of enhancer activity for that KRE, colored by PhyloP evolutionary conservation.

Supplementary Fig 6: Validation of gene editing at Kit response elements. A) Sanger sequencing of PCR-amplified region surrounding the SPRED1-KRE in control (EC) and SPRED1-KRE-targeted cells. B) Sanger sequencing of PCR-amplified region surrounding the DUSP5-KRE in control (EC) and DUSP5-KRE-targeted cells. C) Annotation of transcription factor binding sites at BCL11A, NAB2, DUSP5 and SPRED1 KREs. Motif sequences are color coded. sgRNA target sequences are boxed.

Supplementary Table Legends

1) gRNA sequences for RNP Nucleofection, CRISPRi and CRISPR-KO and primer sequences for qPCR.
2) Source of publicly available ChIP-seq datasets.
3) Dataset of differentially accessible sites between PBS treatment and SCF treatment in Control HUDEP2 cells.
4) Dataset of enriched motifs in Kit Activated regions and Kit Repressed regions.
5) Dataset of TOBIAS Footprinting analysis between PBS treatment and SCF treatment in Control HUDEP2 cells. Footprints upregulated in AP1 and EGR and downregulated in GATA.
6) List of SNPs associated with hematopoietic traits at Kit Activated sites.
7) List of EGR1 sensitive and -insensitive with AP1 and EGR1 occupancies and ATAC-seq peaks assigned by Log 2 BPM values.
8) Dataset of TOBIAS Footprinting analysis for EGR1 Sensitivity (sgControl (+PBS) vs sgEGR1 (+PBS)) and EGR1 Sensitivity to Kit response (sgControl(+SCF) vs sgEGR1(+SCF)) and Co-factors enriched at EGR1 Sensitive KREs.
9) List of features selected for training the XGBoost model.
10) List of differentially expressed genes and common genes in HUDEP2 and CD34+ between PBS treatment and SCF treatment.
11) List of KREs scored and evaluation metrics for XGBoost Model to predict Kit sensitivity in HUDEP-2.
12) List of AP1 and EGR1 occupied sites at KREs.
13) List of KREs scored and evaluation metrics for XGBoost Model to predict Kit sensitivity in MEPs.
14) List of differentially expressed EGR1 sensitive and -insensitive genes.
15) List of EGR1-Sensitive KREs scored and evaluation metrics for XGBoost Model to predict EGR1 sensitivity in HUDEP-2.

16) List of EGR1-Sensitive KREs scored and evaluation metrics for XGBoost Model to predict EGR1 sensitivity in MEPs.

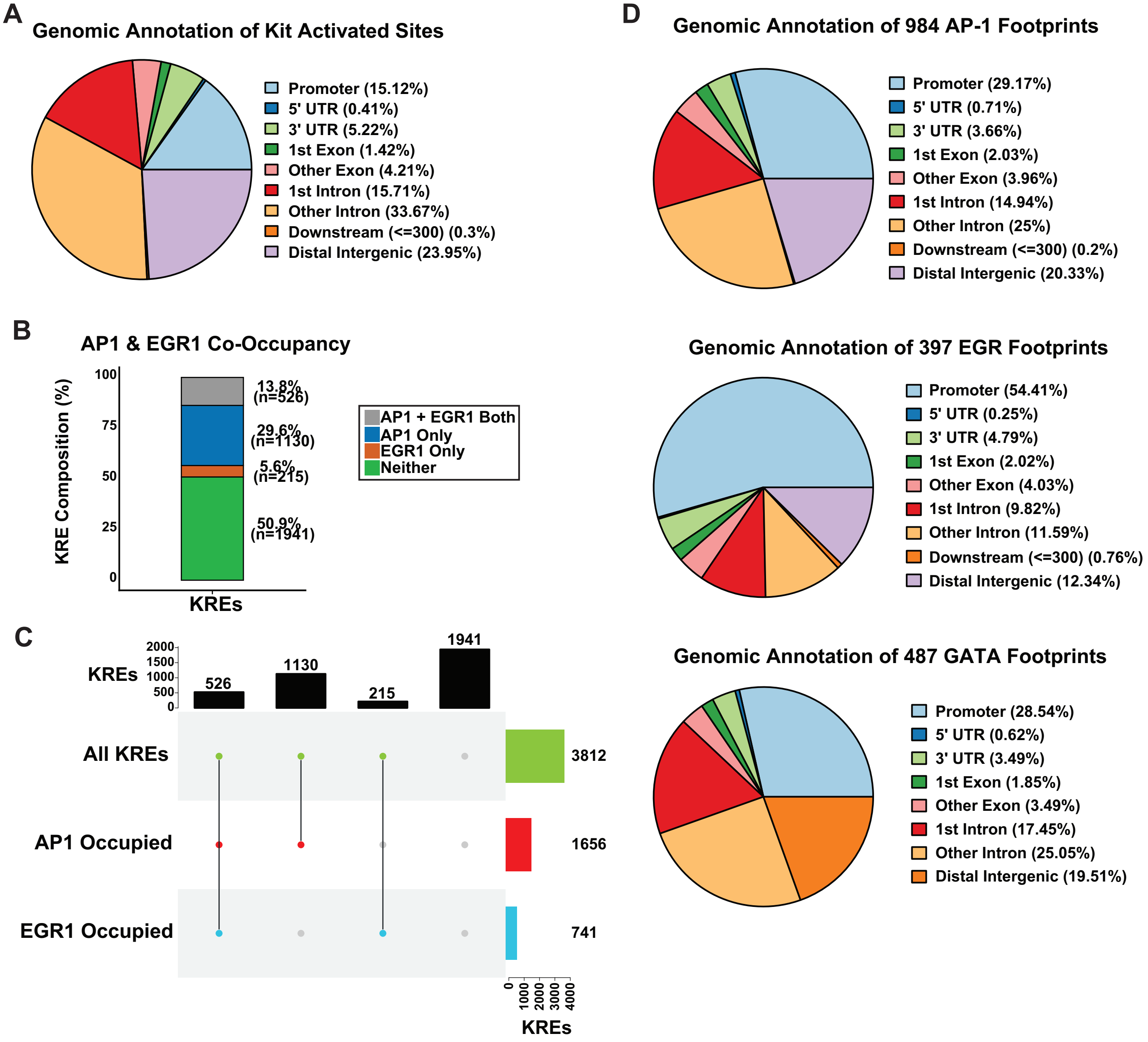
A
Genomic Annotation of Kit Activated Sites
Promoter (15.12%)
5' UTR (0.41%)
3' UTR (5.22%)
1st Exon (1.42%)
Other Exon (4.21%)
1st Intron (15.71%)
Other Intron (33.67%)
Downstream (<=300) (0.3%)
Distal Intergenic (23.95%)
B
AP1 & EGR1 Co-Occupancy
KRE Composition (%)
100
75
50
25
0
13.8% (n=526)
29.6% (n=1130)
5.6% (n=215)
50.9% (n=1941)
KREs
AP1 + EGR1 Both
AP1 Only
EGR1 Only
Neither
C
KREs
2000
1500
1000
500
0
526
1130
215
1941
All KREs
AP1 Occupied
EGR1 Occupied
3812
1656
741
0
1000
2000
3000
4000
KREs
D
Genomic Annotation of 984 AP-1 Footprints
Promoter (29.17%)
5' UTR (0.71%)
3' UTR (3.66%)
1st Exon (2.03%)
Other Exon (3.96%)
1st Intron (14.94%)
Other Intron (25%)
Downstream (<=300) (0.2%)
Distal Intergenic (20.33%)
Genomic Annotation of 397 EGR Footprints
Promoter (54.41%)
5' UTR (0.25%)
3' UTR (4.79%)
1st Exon (2.02%)
Other Exon (4.03%)
1st Intron (9.82%)
Other Intron (11.59%)
Downstream (<=300) (0.76%)
Distal Intergenic (12.34%)
Genomic Annotation of 487 GATA Footprints
Promoter (28.54%)
5' UTR (0.62%)
3' UTR (3.49%)
1st Exon (1.85%)
Other Exon (3.49%)
1st Intron (17.45%)
Other Intron (25.05%)
Distal Intergenic (19.51%)

A

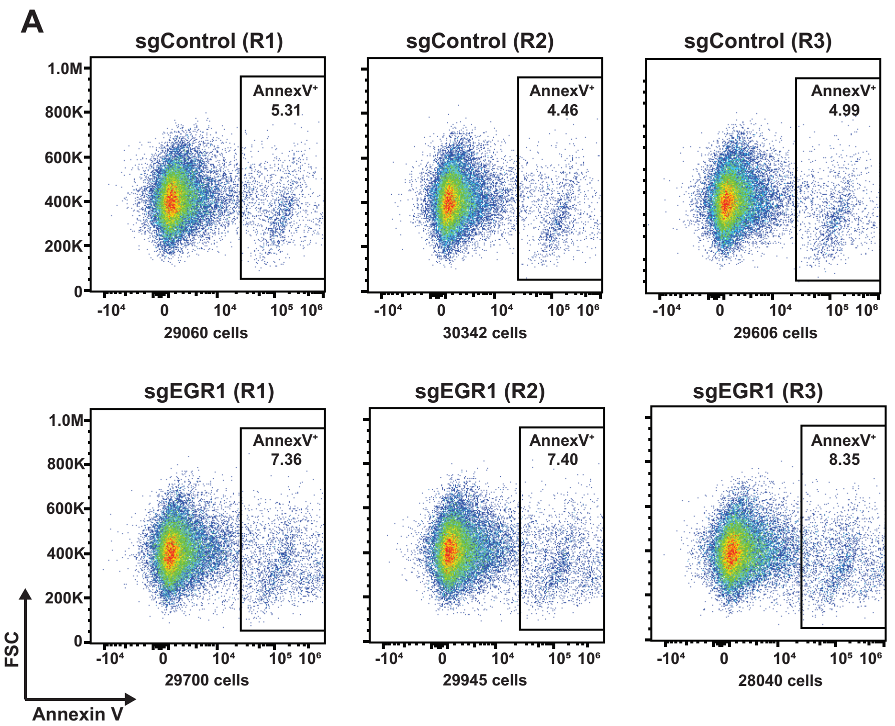

B

sgEGR1 (+SCF/-SCF)	sgControl (+SCF/-SCF) UP	DOWN	NEUTRAL
UP	1441	96	4205
DOWN	65	125	742
NEUTRAL	2306	1237	52804

C

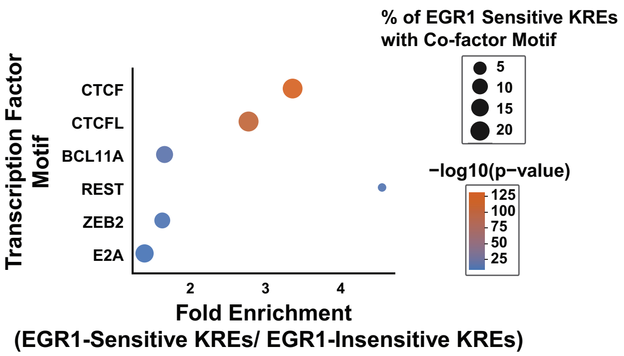

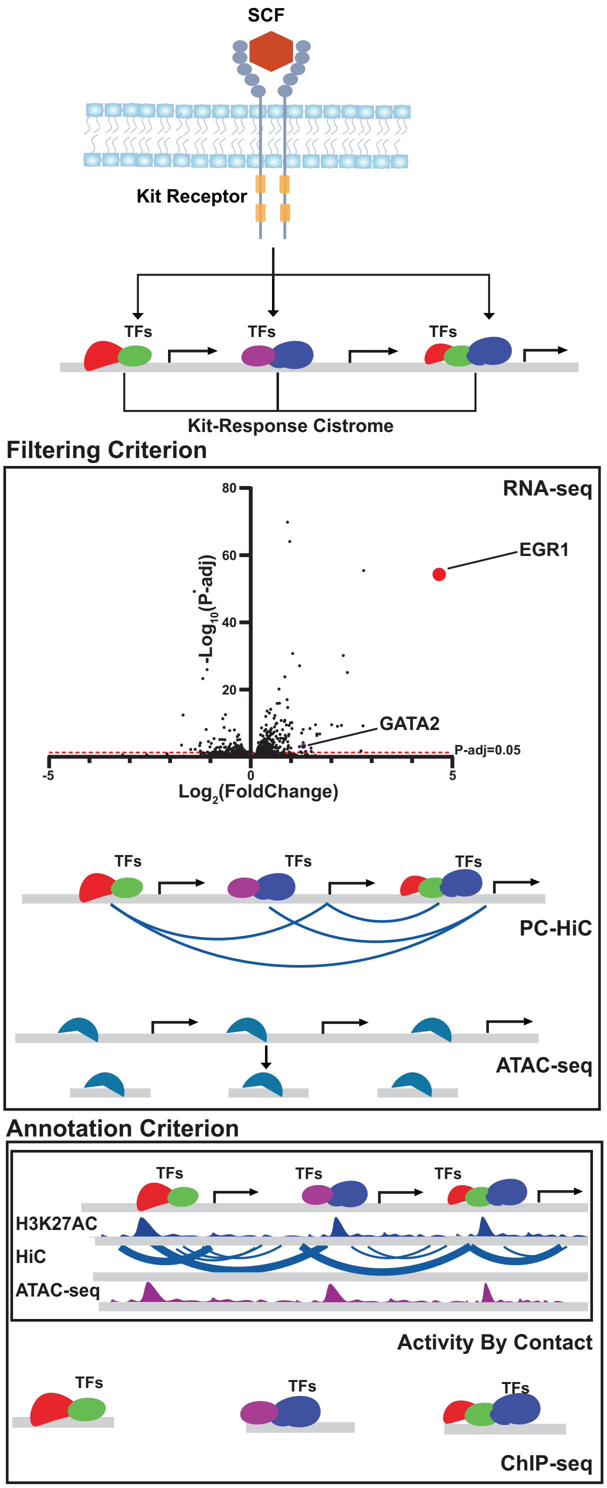
SCF
Kit Receptor
TFs
TFs
TFs
Kit-Response Cistrome
Filtering Criterion
RNA-seq
80
60
40
20
-Log10(P-adj)
EGR1
GATA2
P-adj=0.05
-5
0
5
Log2(FoldChange)
TFs
TFs
TFs
PC-HiC
ATAC-seq
Annotation Criterion
TFs
TFs
TFs
H3K27AC
HiC
ATAC-seq
Activity By Contact
TFs
TFs
TFs
ChIP-seq

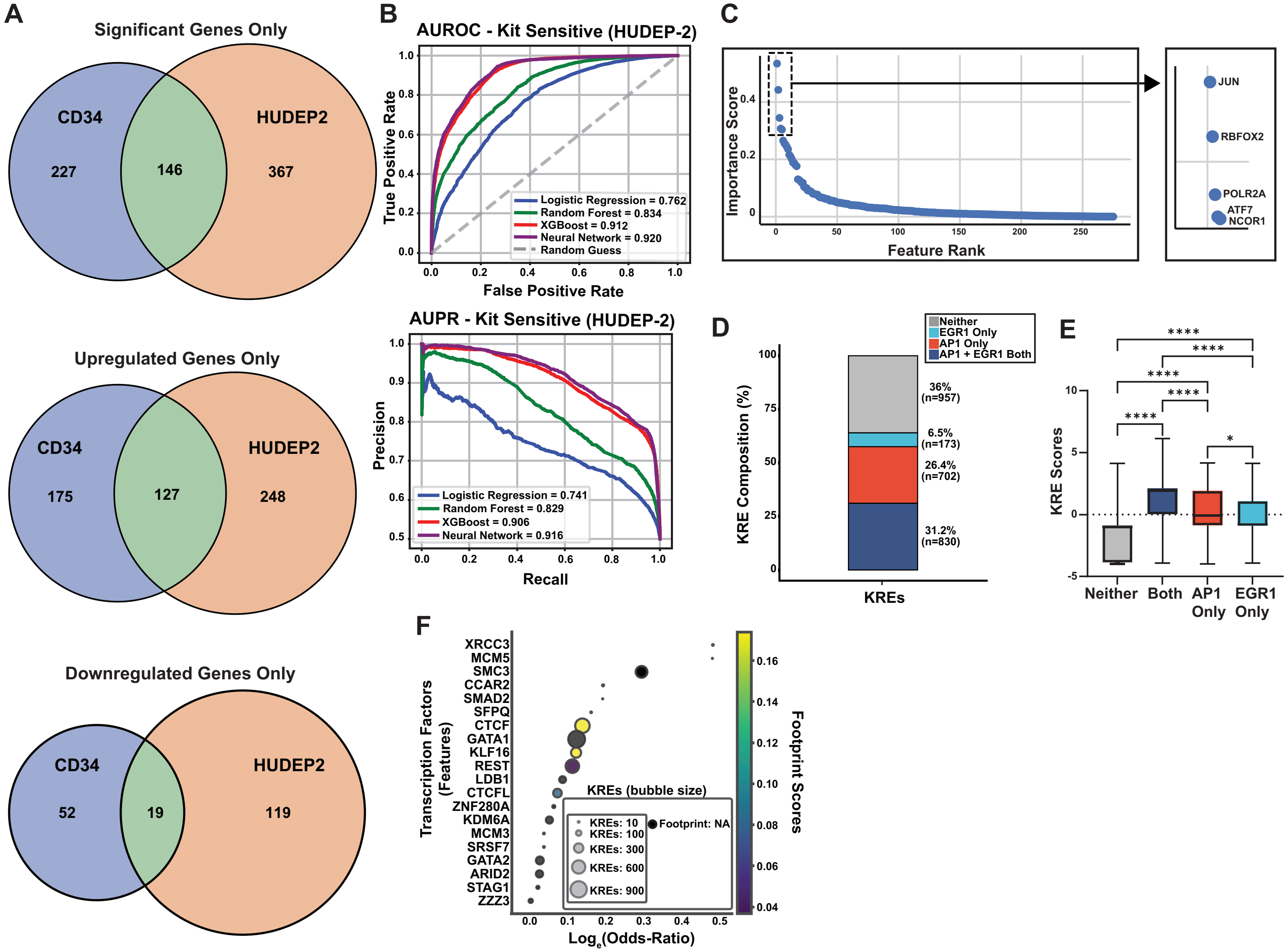
A
Significant Genes Only
CD34
HUDEP2
227
146
367
Upregulated Genes Only
CD34
HUDEP2
175
127
248
Downregulated Genes Only
CD34
HUDEP2
52
19
119
B
AUROC - Kit Sensitive (HUDEP-2)
True Positive Rate
False Positive Rate
Logistic Regression = 0.762
Random Forest = 0.834
XGBoost = 0.912
Neural Network = 0.920
Random Guess
AUPR - Kit Sensitive (HUDEP-2)
Precision
Recall
Logistic Regression = 0.741
Random Forest = 0.829
XGBoost = 0.906
Neural Network = 0.916
C
Importance Score
Feature Rank
JUN
RBFOX2
POLR2A
ATF7
NCOR1
D
Neither
EGR1 Only
AP1 Only
AP1 + EGR1 Both
KRE Composition (%)
36%
(n=957)
6.5%
(n=173)
26.4%
(n=702)
31.2%
(n=830)
KREs
E
KRE Scores
****
****
****
****
****
*
Neither
Both
AP1 Only
EGR1 Only
F
Transcription Factors (Features)
XRCC3
MCM5
SMC3
CCAR2
SMAD2
SFPQ
CTCF
GATA1
KLF16
REST
LDB1
CTCFL
ZNF280A
KDM6A
MCM3
SRSF7
GATA2
ARID2
STAG1
ZZZ3
KREs (bubble size)
KREs: 10
KREs: 100
KREs: 300
KREs: 600
KREs: 900
Footprint: NA
Footprint Scores
Log$_e$(Odds-Ratio)

Dogiparthi et al. Fig S5

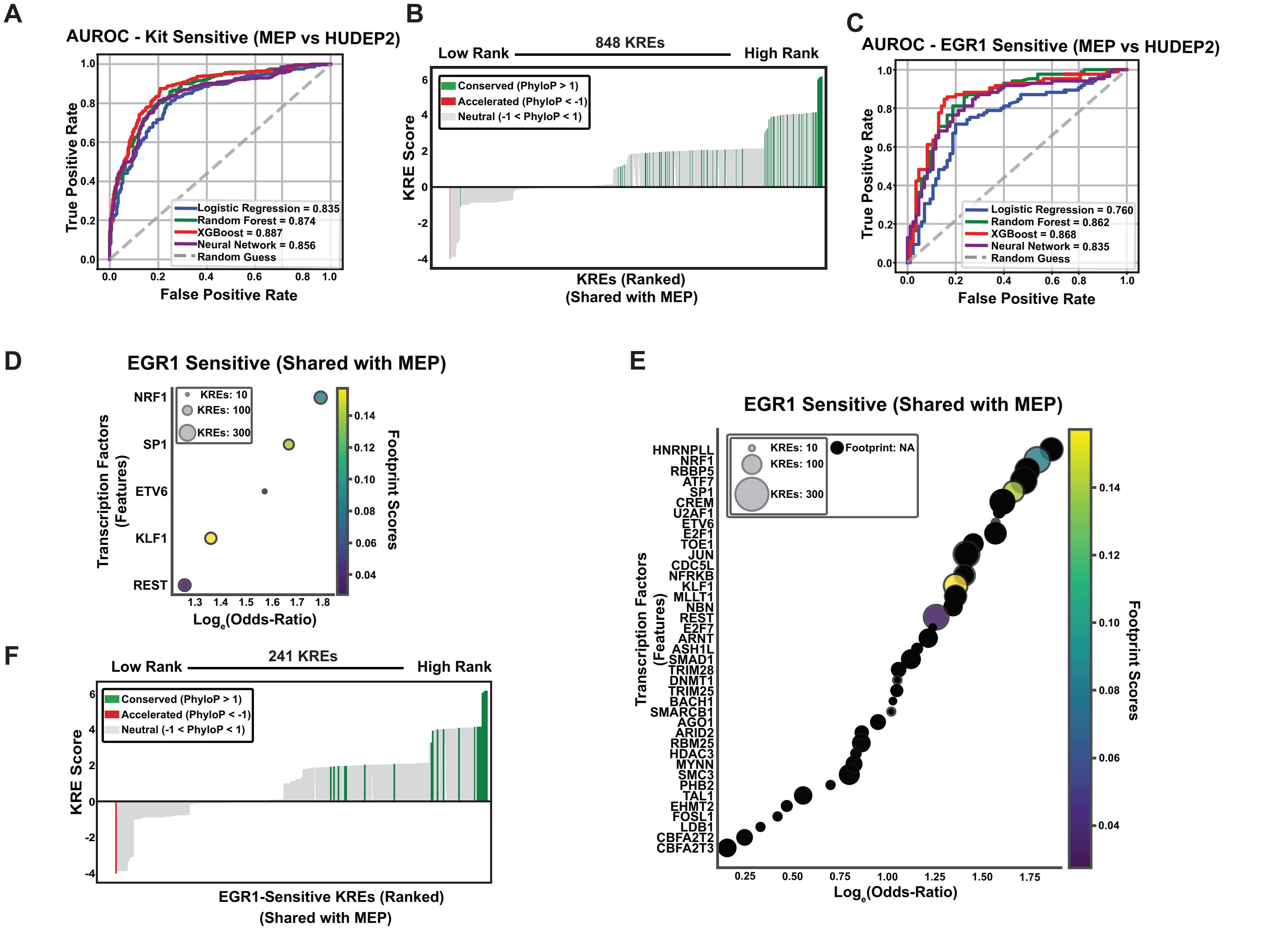

A

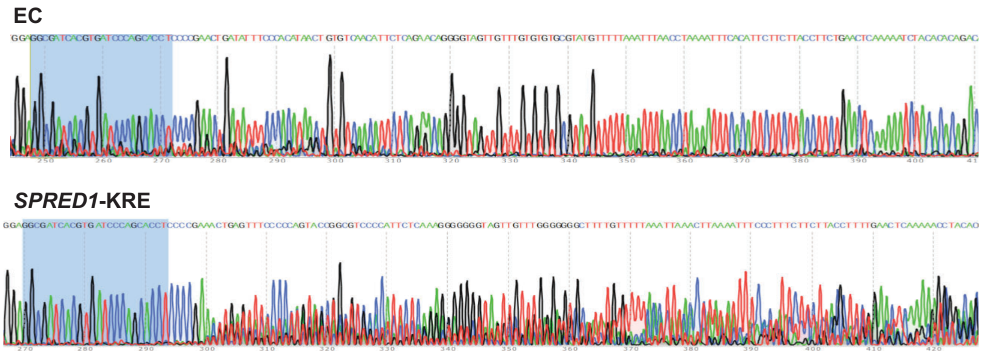

B

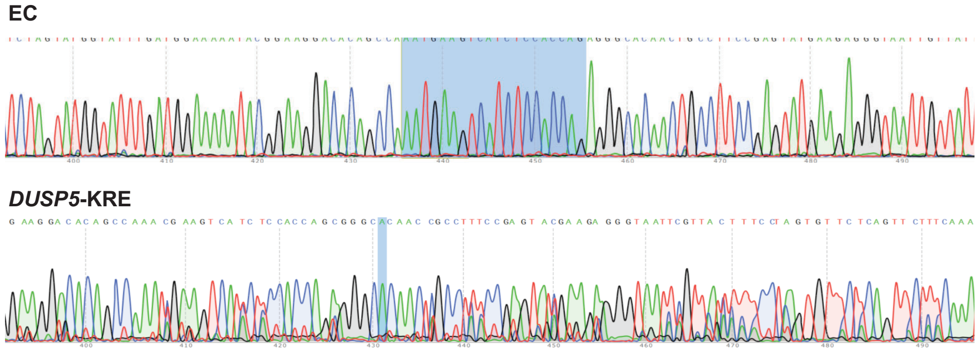

C

- AP-1 Motif
- CTCF Motif
- ETS Motif
- KLF Motif
- EGR, KLF Motifs
- ETS, KLF Motifs

rs35758221 C/A

BCL11A-KRE

ATTAGTCTCCTATTAGTCACTGCTCCTATATGATCCTTTTTCATGTTTTAAAGGAGGATATATATTTTTCTGTTA
CCTTTCCTGACTGTAAAGCAATCACAGGAACCTGCAGGAGGCACTATAAATATGCAAATTTCTGAGAGATAACTG
TTTTCAGTGGTTATGCCTCCAAGTAGTAAGGGAAACAGGAAAGCCGGAGCTTCCTCCTACTTTCAAGTAATTCGT
ATGTTGGTGGTGCCCCCTGCTGCAATCACAGGTTGTCCAAGAAAGGGAGAATAAAATTCAGAAGCATTTAGATCT
GGTTAAT

NAB2-KRE

GGGATGGGGATGCCCAGCTTGGAAGCAGAGGAGCCCAAAGCAGTGATGCTTTGTGTGTGGAAGGGG
GAAGAGGGAGTGGGCAGGCGGAGGGCCGGGCAGCCGCTGGGCTGAGAGTGACGCAGCAGACAAAGC
CACCAACCCTGGCTTGGTATTTTTAAACTGTGCGTGGGTGGGAAGTGGGGGCGGGGACTGGAAGGG
GGGCTCTTCTTGAGGGAGGAGCTCGGAGGCTGGCTTCCTGTTCTCCCTCTGATTTGGCTACCAAAA
AACTTGGGGGGCCATGGTAGAGTGGCCTGGGAG

SPRED1-KRE

GACACGTCCAATATTCACAA -450bp → ACTTTTTAAAGTCTGTACCGAGTCCTGTTTAACGTAAAATAGCTAGAGAAAGGCCAAACAACATTTCCTCCTCCCT
TACTCCCTCCCCACCCCAAAACACACGCTTCTGCTGAGCGGGTGTAGAAGGAGGTACATTGCTGCAGTGTCAGTGC
AGTGTAGAAGCAGAAGTGGCAGCCATGTTCTGTCTCTTTTTGTGAATCGCCCTCATTTGCATAGCACACTCTTCAT
TCATAAAAAAAATAATTAAACATCCATCACCTTGGACATCTTGAAAGGATTTCCCAAGAAAAAAAATTCGCGGCTT
ATTTGTCTGTGTGTAGATTTTTGAGTTCAGAAGGTAAGAAGAATGTGAAATTTTAGGTTAAATTTAAAAACATACG
CACACACAAACAACTACCCCTGTTCTGAGAATGTTGACACAGTTATGTGGAAATATCAGTTCGGGGAGGTGCTGG
GATCACGTGATCGCCTCCATTCATAAAATACCTGGCTGTCCATTCAGAGTGCAGTACAGTCTCTCCCTGCCTTCTG
CAGATTAGACCTACTTTGAGGGAGATCAAGAAGTCTATCTCAGATAATGGTTATTTAGGAGAAGAACTGCTAAAAA
TGCTGAACCGAATGAC

DUSP5-KRE

CCAGCTAATCTGATGGGAACAACATAACCTGATTAAACTAAATTGTTTTGACAGCGTGGCCATTTGAAGAACTGA
GAACACTAGGAAAATAACAATTACCCTCTTCATACTCGGAAGGCAGTTGTGCCCTCTGGTGGAGATGACTTCATT
TGGCTGTGTCCTTCCGTATTTTTCCATCAAATACCATACTAGAGGAAGTTTCTCCGTGGAGATTACTTTACAGAC
TTTTAGAAGTCC
